# Supplementary material for: A Brain-Targeted Approach to Ameliorate Memory Disorders in a Sporadic Alzheimer’s Disease Mouse Model via Intranasal Luteolin-Loaded Nanobilosomes
Source: Pharmaceutics. 2022 Mar 5;14(3):576. doi: 10.3390/pharmaceutics14030576 (PMC8950550; doi:10.3390/pharmaceutics14030576)
Supplement: Supplementary file 1 [file pharmaceutics-14-00576-s001.zip › pharmaceutics-1608085-supplementary.pdf]

pharmaceutics

Supplementary Materials: A Brain-Targeted Approach to Ameliorate Memory Disorders in a Sporadic Alzheimer's Disease Mouse Model via Intranasal Luteolin-Loaded Nanobilosomes

Manal A Elsheikh, Yasmin A. El-Feky, Majid Mohammad Al-Sawahli, Merhan E. Ali, Ahmed M. Fayez and Haidy Abbas

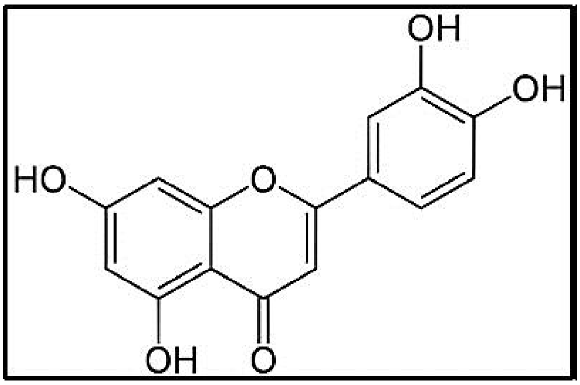

Figure S1. Chemical structure of luteolin.
